# Marrow Microenvironmental Pathobiology and Therapeutic Opportunities for *TP53*-Mutated Myelodysplastic Syndrome/Acute Myeloid Leukemia

**DOI:** 10.3390/cancers18020275

**Published:** 2026-01-16

**Authors:** Cameron J. Hunter, Annie P. Im, Rory M. Shallis

**Affiliations:** 1 Division of Malignant Hematology and Medical Oncology, Department of Medicine, UPMC Hillman Cancer Center, Pittsburgh, PA 15232, USA; huntercj4@upmc.edu (C.J.H.);; 2 Department of Malignant Hematology, H. Lee Moffitt Cancer Center, Tampa, FL 33612, USA

**Keywords:** myelodysplastic syndrome, acute myeloid leukemia, *TP53*, bone marrow microenvironment, chemoresistance, immunosuppression

## Abstract

Mutations in *TP53* inhibit p53 protective behaviors including cell cycle arrest, DNA damage repair protein recruitment, and apoptosis. The ubiquity of p53 in genome-stabilizing functions leads to an aberrant tumor microenvironment in *TP53*-mutated myelodysplastic syndrome (MDS) and acute myeloid leukemia (AML). Profound immunosuppression mediated by myeloid-derived suppressor cells, the upregulation of cytokines and cell-surface receptors on leukemic cells, the suppression of native immune regulator cells, and metabolic aberrations in the bone marrow are features of the *TP53*-mutated AML/MDS marrow microenvironment. These localized changes in the bone marrow microenvironment (BMME) explain why traditional therapies for MDS/AML, including chemotherapeutics and hypomethylating agents, are not as effective in *TP53*-mutated myeloid neoplasms and demonstrate the dire need for new treatments in this patient population. The unique pathophysiology of *TP53*-mutated disease also provides new therapeutic approaches which are being studied, including intracellular targets (MDM2, p53), cell-surface protein biologics (immune checkpoint inhibitors, BiTE therapy, and antibody–drug conjugates), cell therapies (CAR-T, NK-cell), signal transduction pathways (Hedgehog, Wnt, NF-κB, CCRL2, and HIF-1α), and co-opted biologic pathways (cholesterol synthesis and glycolysis). In this review, we will discuss the pathophysiologic anomalies of the tumor microenvironment in *TP53*-mutant MDS/AML, the hypothesized mechanisms of chemoresistance it imparts, and how novel therapies are leveraging diverse therapeutic targets to address this critical area of need.

## Simple Summary

The marrow microenvironment associated with *TP53*-mutated myelodysplastic syndrome/acute myeloid leukemia is one of immunosuppression, namely mediated by myeloid-derived suppressor cells and characterized by aberrancy of native immune regulator cells, cytokines, leukemic cell-surface receptors and cellular metabolism. The growing understanding of this pathobiology with the molecular subset of disease has fostered the development of novel therapies that leverage intracellular/signal transduction targets, co-opted biologic pathways, and cell-surface elements that impact the immune environment for this population in critical need of effective therapeutic strategies.

## 1. Introduction

*TP53* encodes for the tumor-suppressor p53 protein, which plays an essential role in genomic stability [1,2]. Once DNA damage is detected by one of three DNA damage response (DDR) kinases (ATM, ATR, and DNA-PK), p53 is activated and protects the cell in three distinct ways [3,4]. It mediates cell cycle arrest (primarily through p21), activates a slew of DNA damage repair proteins, and initiates apoptosis if the damage is deemed to be too extensive or irreparable [4,5]. *TP53* inactivation (via overexpression of MDM2, a negative regulator of *TP53*) or direct mutation impairs these functions, causing unregulated proliferation and immune evasion [6,7,8,9,10].

Mutations in *TP53* are detected in nearly half of all human malignancies that have been extensively sequenced [11,12,13]. While *TP53* is mutated less often in myeloid neoplasms (~10%) [14,15,16,17], there is enrichment among patients who are older or incur disease arising out of prior cytotoxic therapy, previously known as therapy-related AML, which associated with a 30–40% rate of *TP53*-mutated disease [18,19,20,21]. Evidence of age-related *TP53*-mutated clones preferentially expanding under the pressure of cytotoxic therapy, rather than evidence showing prior therapy directly inducing new *TP53* mutations eventually begetting disease, has been proffered [22]. Ultimately, the chemoresistance exhibited by *TP53*-mutated MDS/AML associates with lower rates of remission, shorter durations of remission, and, consequently, a dismal median overall survival (OS) of 5–10 months relative to other etiologies of MDS/AML, which often have a median OS of >24 months [19,23,24]. Recent studies have also shown that biallelic *TP53* alteration does correlate with poorer prognosis when compared to monoallelic loss, suggesting a tiered effect on prognosis based on the amount of available native protein [25,26].

These notably poorer outcomes have led recent classification systems and guideline bodies to recategorize *TP53*-mutated disease as a unique entity. The most recent World Health Organization (WHO-5) classification concedes that MDS with biallelic *TP53* inactivation (MDS-bi*TP53*) may be regarded as a functional AML equivalent for therapeutic considerations [27]. Additionally, the 2022 International Consensus Classification (ICC) considers *TP53*-mutated myeloid neoplasms to be a separate category independent of blast percentage at diagnosis due to their universal aggressiveness/treatment resistance [28,29,30,31].

The bone marrow microenvironment (BMME) is an encompassing term for dynamic multicellular marrow niches that regulate the function and differentiation of hematopoietic stem/progenitor cells (HSCs) [32,33,34]. These niches contain structural cells (stromal cells, endothelial cells, and osteoblasts), immune regulatory cells (macrophages, regulatory T cells) [35,36], and are influenced by specific cytokines (CXCL12, SCF, and TGF-β) that can allow the selective expansion of hematopoietic cell lines and maintenance of homeostasis [37,38]. Further signaling via the HIF-1α/CXCR4 pathway leads to stabilization and maturation of the BMME niche [39,40]. Aberrations in any component of this delicate homeostasis can be leukemogenic. Studies have found that cellular dysfunction leading to changes in gene (SBDS) or cytokine (β-catenin) expression can precipitate myeloid neoplasms [41,42,43,44]. *TP53*-mutated myeloid malignancies, specifically, are characterized by the overexpression of suppressive regulatory T cells, myeloid-derived suppressor cells (MDSCs), and the underexpression of surveilling cytotoxic/helper T cells and NK cells [45,46], ultimately subject to an extremely immunosuppressive BMME.

In this review, we will discuss the pathophysiologic anomalies of the tumor microenvironment in *TP53*-mutant MDS/AML, the hypothesized mechanisms of chemoresistance it imparts, and how novel therapies are leveraging diverse therapeutic targets to address this critical area of need.

## 2. p53 Protein Function and Aberrancy with Mutation

**Figure 1:**
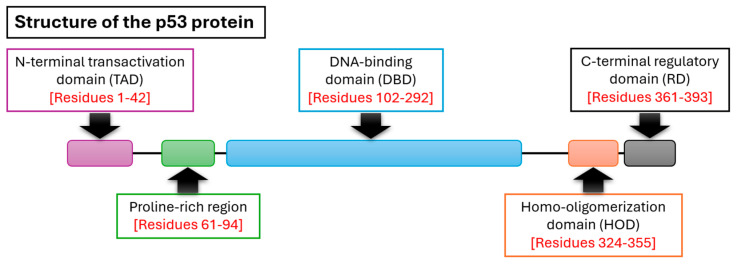
The main domains of the p53 protein.

The fully translated p53 protein consists of five key domains [Figure 1]. An N-terminal transactivation domain, which is the site of MDM2 binding [47,48]; a proline-rich domain, which mediates nuclear export and apoptosis via MAPK signaling [49]; the primary DNA-binding domain (DBD), which is critical for its genome surveillance activity [50,51]; the homo-oligomerization domain (HOD), which helps form the biologically active tetrameric form of p53 [52]; and the C-terminal domain, which modulates the DBD [53,54].

Mutations in *TP53* are most common in the DBD (~85%), of which around 10% are frameshift or nonsense mutations preventing protein production entirely, with the remaining 90% being missense mutations leading to non-functional protein [55,56,57]. These missense mutations have been found to exert a dominant-negative effect in myeloid neoplasms [58,59]. A minority of *TP53* mutations occur in the HOD (~10%), which also exerts a dominant-negative effect [60]. The inactivation of p53 leads to impaired genomic surveillance and the suppression of many downstream signals which would otherwise halt reproduction or signal for apoptosis, creating a microenvironment of profound immunosuppression.

While *TP53* mutations are inherently oncogenic, the transition to myeloid neoplasm is driven by a selective pressure. The most common of these are clonal hematopoiesis or the receipt of cytotoxic chemotherapy. Clonal hematopoiesis has been found to drive hematopoietic stem cell (HPSC) expansion on its own, specifically through interaction with the epigenetic regulator EZH2 [61,62]. Cytotoxic chemotherapy has been found not to directly induce *TP53*-mutant myeloid neoplasm but rather the inherent chemoresistance of these cells leads to preferential expansion after exposure to chemotherapy [22,63].

**Figure 2:**
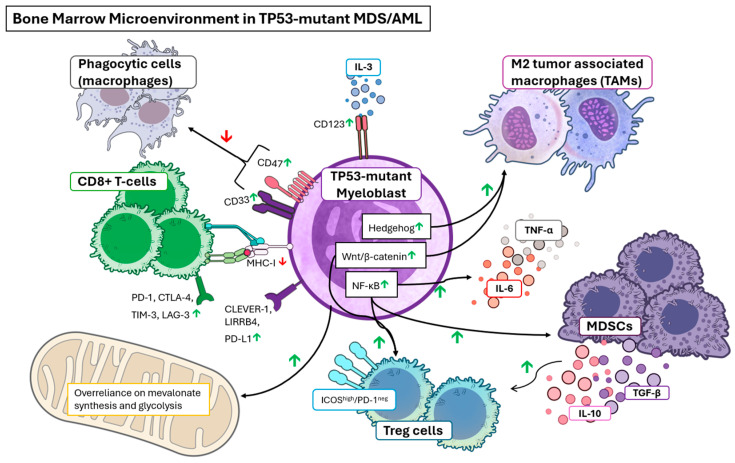
Elements of the bone marrow microenvironment in *TP53*-mutated MDS/AML [195]. The combination of upregulating immunosuppressive M2 macrophages, MDSCs, and Treg cells while downregulating native macrophages and cytotoxic T cells create the profoundly immunosuppressive BMME.

These many modulations of the *TP53*-mutant BMME contribute to the resistance to many conventional cancer-directed therapies, including anthracycline/cytarabine [64,65], hypomethylating agents (decitabine/azacitidine) [66], and venetoclax [67,68,69]. Consequently, a valid emphasis has been placed on the identification of viable therapeutic strategies bypassing classical cytotoxic strategies for *TP53-*mutated MDS/AML. Below are the major mechanisms of chemoresistance imparted by *TP53*-mutated MDS/AML [Figure 2] and the state of research into therapeutics leveraging BMME aberrancy [Table 1].

## 3. *TP53*-Mutated MDS/AML Marrow Microenvironmental Features and Therapeutic Implications

### 3.1. Leukemic Cell Modifications

#### 3.1.1. Direct Effect of the Mutant p53 Protein

Targeting non- or sub-functioning mutant p53 proteins and, namely, restoring cellular pro-apoptotic functioning amongst malignant clones is the ideal downstream strategy. Two general classes of agents are hypothesized to exert this effect: MDM2 inhibitors and p53 “refolding agents”. MDM2 binds to p53 and marks it for degradation with the thought that MDM2 inhibition would lead to higher levels of native p53 protein [70]. For MDS/AML, MDM2 inhibitors have not advanced beyond clinical trials, with the most notable study being the MIRROS Phase III trial, which analyzed cytarabine with or without the small-molecule MDM2 inhibitor idasanutlin [71]. Of note, this trial eventually enrolled patients irrespective of *TP53* status, as some missense mutations predict retained p53 functionality. This trial was stopped for futility with no difference in the median overall survival (mOS) or CR rate, though the combination did improve the overall response rate (ORR). A Phase I/II study of the MDM2 inhibitor alrizomadlin is actively recruiting [72]. Challenges to the implementation of these agents include profound myelosuppression, and case studies suggest a risk of *TP53*-altered hematopoiesis after exposure to these agents [73,74,75,76].

A more promising strategy for *TP53*-mutated MDS/AML is agents which induce p53 refolding to its native, biologically active conformation [77]. The most studied agent in this class is APR-246 (eprenetapopt), for which a Phase III trial comparing azacitidine ± APR-246 for patients with *TP53*-mutated MDS was recently completed and was negative for its primary endpoint of complete remission, although secondary endpoint data are pending [78]. The viability of APR-246, or in the oral form APR-548, in a subsequent *TP53*-mutated myeloid malignancy trial with modified trial design is currently being explored. Arsenic trioxide (ATO) was recently found to directly bind and rescue a number of *TP53* structural mutations [79] and was found to induce AML cell death in vitro [80]. The results of several currently active prospective trials of ATO in *TP53*-mutated MDS/AML are pending, with other prospective trials being planned.

#### 3.1.2. Inhibition of T-Cell Activation

TP53-mutated MDS/AML marrow tissue is shown to be that of downregulated major histocompatibility complex (MHC) class I and II expression and upregulated programmed death-ligand 1 (PD-L1) expression [81,82,83]. T-cell activation is predicated upon dual signals [84], one between the T-cell receptor (TCR) and MHC (CD4+ helper T cells binding to MHC class II on antigen-presenting cells and CD8+ cytotoxic T cells binding to MHC class I on tumor cells [82,85], also requiring co-stimulation between OX40-OX40L and 4-IBB-4-IBBL in order to fully activate [86]) and another one most often between CD28 on the T cell and CD80/86 on the tumor cell [87]. These two signals lead to the proliferation of countless T cells that recognize the presented antigen but are mediated by the immune checkpoints like CTLA-4 in the T cell, which competes with CD28 for binding to CD80/86 and downregulates the immune response [88]; as well, the binding between programmed cell death protein-1 (PD-1) on cytotoxic T cells and PD-L1 on tumor cells inhibits the cytotoxic activation [89]. Further upregulation of PD-L1 is noted in patients with myeloid neoplasm who were treated with hypomethylating agents (HMAs) decitabine or azacitidine [90,91].

In light of the impressive results of ICIs for other cancers like melanoma and lung cancer [92,93], it has been an area of considerable research interest in MDS/AML [94]. Their importance is further highlighted given the upregulation of immune checkpoint proteins found with *TP53*-mutated MDS/AML [83]. However, the majority of trials have not been specific to *TP53*-mutated disease. Amongst classical ICIs targeted against PD-1, PD-L1, or CTLA-4, Phase II and III trials of pembrolizumab [95,96], nivolumab [97], durvalumab [98], and ipilimumab [99] have failed to show survival benefit. Secondary analyses are ongoing and may hopefully identify biomarkers useful for future trial design with classical ICIs.

Non-classical ICIs are also being investigated. Myeloid-specific immune checkpoints including LIRRB3/LIRRB4 have been discovered to be overexpressed in *TP53*-mutated MDS/AML and have correlated with enhanced leukemic cell survival [91,100,101]. These alterations help *TP53*-mutated leukemic MDS/AML evade detection by cytotoxic T cells and create a profoundly immunosuppressed environment. Overstimulation of other proteins has been found to play a role in T-cell inhibition as a mechanism of leukemic proliferation, including CTLA-4, TIM-3, and LAG-3 [102,103,104,105]. Beyond T cells, studies have shown that monocyte immune checkpoints also play a role in the proliferation of MDS/AML, including Clever-1, and their blockade can attenuate the effect of other therapies [106,107]. Trials evaluating non-classical immune checkpoint inhibition including TIM-3 and LAG-3 have either yielded no positive results or are ongoing [108,109]. Clinical data for non-classical immune checkpoint inhibition LILRB4 has emerged as an attractive myeloid-specific immune checkpoint target, with Phase I clinical trials as a monoclonal antibody (IO-202) or STAR-T cell forms showing promising results [110,111]. Studies of the monocyte-specific ICI, bexmarilimab, show promising results for *TP53*-mutated MDS [112].

#### 3.1.3. Anti-Phagocytic Activity

Another major mechanism contributing to the immunosuppressive environment in TP53-mutated myeloid neoplasm is the overexpression of “don’t eat me” cell-surface proteins preventing phagocytosis. One major example is between CD47 on cancer cells and SIRP alpha on phagocytic cells [113,114]. This protein is heavily overexpressed on cancer cells to evade immune detection and phagocytosis by macrophages [115,116], although expression varies based on genotype [117]. The anti-CD47 antibody magrolimab had promising Phase II results, leading to the development of a trio of Phase III trials. The ENHANCE trial compared azacitidine ± magrolimab, the ENHANCE-2 trial compared azacitidine + magrolimab versus azacitidine/venetoclax or intensive chemotherapy, and the ENHANCE-3 trial compared azacitidine/venetoclax ± magrolimab [118,119,120]. Each of these trials were discontinued due to futility in the experimental arm.

CD33 is another important protein that prevents detection by macrophages and is notably overexpressed on AML leukemic blasts and appears dispensable for hematopoiesis, allowing for a favorable therapeutic index [121,122]. Gemtuzumab ozogamicin (GO), an antibody–drug conjugate (ADC) targeting CD33, received FDA approval in 2017 for adult and pediatric patients with CD33+ AML [123,124]. However, many other CD33-directed therapies have been investigated since. Several Phase I trials studying CD33+ CAR-T/CAR-NK cells are ongoing [125,126], and preclinical data for a CD33-targeting ADC with a GSPT1-targeted payload has shown striking leukemia cell kill in *TP53*-mutated cell lines [127].

#### 3.1.4. Enhancing Leukemic Cell Proliferation

*TP53*-mutated MDS/AML exhibits further chemoresistance by the upregulation of cell-surface proteins that aid in leukemic cell proliferation. One protein that is of particular interest is CD123, the receptor for IL-3, which leads to downstream proliferation [128,129]. CD123 is overexpressed in MDS/AML cells, but the extent of overexpression varies based on the cytogenetic profile of the disease [130,131]. Monoclonal antibodies targeting CD123 have not proven to be overtly successful yet, with the Phase II/III trial comparing decitabine ± talacotuzumab showing no survival benefit with considerable toxicity [132]. After this result, other Phase II studies of single-agent talacotuzumab were terminated early [133,134]. CD123-CD3 bispecific T-cell engagers (BiTEs) have also been studied in patients with MDS/AML harboring *TP53* mutations. A Phase II trial of flotetuzumab was terminated early [135], although a Phase II trial of vibecotamab associated with a 50% CR_L_ rate amongst patients with *TP53*-mutated disease [136]. Administration schedules have also limited enthusiasm for some of these earlier products. Another CD123-targeted agent, tagraxofusp (diphtheria toxin-IL-3 conjugate), is actively recruiting for a Phase II trial and has demonstrated encouraging data in combination with HMA + venetoclax for newly diagnosed AML [137]. Other CD123-targeted therapies in early trials include CD123-CD16 BiTE AFM28 [138], the ADC pivekimab sunirine (IMGN632) [139], and CAR-T/CAR-NK cell trials [140,141].

### 3.2. Abnormal Immune Cell Populations

In addition to aberrant T-cell surface protein expression, T-cell populations are inherently abnormal in *TP53*-mutated MDS/AML. One study found elevated levels of ICOS^high^/PD-1^neg^ T-regulatory cells in patients with *TP53*-mutated MDS/AML [46]. Consistent with prior research, this study also found that the presence of more of these aberrant T-regulatory cells correlated with a poor prognosis independent of TP53 mutation status [142,143]. Furthermore, effector memory T cells are also overrepresented in TP53-mutated MDS/AML and overexpress genes that drive exhausted T-cell states, including PRDM1, leading to a less effective immune response [144].

Furthermore, chronic inflammatory states like cancer are subject to disrupted regular myelopoiesis, diverting immature myeloid cells from monocytes/granulocytes to produce more MDSCs, which lack phagocytic activity and are immunosuppressive and anti-inflammatory in nature [145,146]. MDSCs employ several pathways to mediate immunosuppression including their expression of the protein-inducible NOS and COX2 [147,148,149,150], their production of the cytokines TGF-β and IL-10 [151,152], and the induction of regulatory T cells amongst the larger T-cell repertoire [153]. MDSCs are found to be highly overexpressed in myeloid neoplasms, and elevated levels confer a poor prognosis [154,155,156,157]. AML marrows are also found to be preferentially enriched for M2 macrophages. Compared to their pro-inflammatory M1 macrophage counterparts, M2 macrophages are anti-inflammatory cells that aid in phagocytosis resistance among leukemic stem cells [158]. One study of tumor-infiltrating leukocytes in AML marrows demonstrated that *TP53*-mutated AML associates with a high expression of CD206, a marker of M2 macrophages [159]. No therapies directly targeting these immunosuppressive cell populations are currently in clinical trial.

### 3.3. Cytokine Signaling Pathways

Wnt/β-catenin signaling leads to stem cell proliferation, and overactivation of this pathway is associated with a higher risk of relapse and poorer prognosis with myeloid neoplasms [160,161], while its disruption leads to decreased proliferation [162]. Studies have also shown that Wnt signaling leads to increased tumor-associated macrophages and T-regulatory cell populations while decreasing cytotoxic T-cell activity in the BMME [163,164]. Upregulation of Wnt signaling in *TP53*-mutated MDS/AML has been associated with an improved rate of response to CAR T-cell therapy [165]. However, this pathway is not currently undergoing trials specific to *TP53*-mutated MDS/AML [166].

The Hedgehog pathway is also altered in MDS/AML and plays a major role in cell differentiation. It is upregulated in many cancers, including AML [167,168]. This signaling causes increased levels of PD-L1 overexpressing tumor-associated macrophages, which leads to T-cell exhaustion and inhibits the anti-tumor inflammatory response [169,170]. However, studies found that TP53-mutated MDS/AML has lower rates of Hedgehog expression compared to their TP53-WT counterparts and had a consequently poorer response to Hedgehog-directed therapy [171,172], making it a poor target for *TP53*-mutated MDS/AML. This was validated in the Phase III BRIGHT AML 1019 trial evaluating glasdegib (smoothened inhibitor), which failed to show improvement in mOS for the entire cohort, as well as for the *TP53*-mutated subset [173].

Dysregulation of NF-κB signaling is found in up to 40% of AML patients, contributing to oncogenesis and chemoresistance [174]. It does so through two main mechanisms: upregulation of pro-inflammatory cytokines like IL-6 and TNF-α, as well as increasing populations of the aforementioned T-regulatory cells and MDSCs. These aberrations lead to a very inhospitable BMME for traditional immune cells and prevent detection. Pathologic NF-κB signaling is additionally associated with upregulation of the anti-apoptotic BH3 proteins BCL-XL and MCL-1, which promotes leukemic stem cell chemoresistance [175,176]. Bortezomib is a proteasome inhibitor that acts on the NF-κB signaling pathway. It advanced to a Phase III trial, which did not improve treatment outcomes for children with AML, and has not been studied in adults [177].

The CCRL2/IFN-γ pathway is upregulated in *TP53*-mutated AML, especially acute erythroid leukemia. This pathway is upregulated in the absence of viable p53 proteins and has been associated with transforming pre-leukemic single-hit *TP53* clones into multi-hit *TP53*-mutated AML [178]. Attempts have been made to target this pathway, including with a pyroolobenzodiazepine-conjugated anti-CCRL2 ADC [179].

Lastly, the HIF-1α/CXCR4 pathway is upregulated in *TP53*-mutated MDS/AML. Preclinical studies of the HIF-1α inhibitor echinomycin have shown promise, specifically for myeloid malignancies, but have not reached clinical trials [180]. The CXCR4 inhibitor plerixafor showed higher survival among the *TP53*-mutated subgroup in a Phase I trial, although this result was underpowered to reach statistical significance [181].

### 3.4. Other Biological Pathways

Amongst the natural pathways leveraged by MDS/AML cells to proliferate is the cholesterol synthesis pathway. Xia et al. found that lovastatin (an HMG-CoA reductase inhibitor) can induce a significant apoptotic effect in human AML cells through the inhibition of geranylgeranylation [182]. Another study found that statins induce the rapid differentiation of AML cells leading to apoptosis [183]. Bisphosphonates exhibit anti-leukemic activity by blocking farnesyl pyrophosphate synthase, an enzyme further downstream in the mevalonate pathway [184]. Subsequent studies have identified TP53-mutated MDS/AML as being reliant upon the mevalonate pathway for an adaptive stress response, upregulating glutathione production for the management of reactive oxygen species (ROS), and leading to cytarabine chemoresistance [185]. Given the reliance of leukemic cells on cholesterol synthesis for replication, statins have been explored both in Phase I/II trials without efficacy results being reported [186,187].

Many malignancies are characterized by an overreliance on glycolysis for energy production, given that they proliferate rapidly in anaerobic conditions, preferentially converting glucose to lactate, termed the Warburg Effect [188]. Hematologic malignancies are no exception, demonstrating that the upregulation of genes involved in glycolysis and glucose transport correlate with reduced chemosensitivity and poorer OS [189,190,191]. Additionally, one study found that Vitamin D leads to a precipitous increase in fructose-1,6-bisphosphatase (*FBP1* gene), the main rate-limiting enzyme in gluconeogenesis, which also suppresses glycolysis and could prohibit leukemic cells from utilizing this energy source [192]. Despite the theoretical benefit from targeting the glycolysis/gluconeogenesis pathways, no trials are currently active.

Lastly, the creatine kinase pathway has been implicated as a target for some patients with activating mutations in the *EVI1* gene. This activating mutation leads to CKMT1 expression and energy production in leukemic cells [193,194]. However, this pathway is not as active in patients with other driver mutations, including *TP53*.

## 4. Conclusions and Future Directions

*TP53*-mutated MDS/AML is among the most complicated to treat due to the many adaptations to the BMME, which cause resistance to many commonly used therapies. While the mechanisms of resistance are still being elucidated, it is suspected that these adaptations make the BMME inhospitable for native immune cells, decreasing the efficacy of immunotherapies and targeted therapies which have seen benefit with *TP53* WT disease. This harsher microenvironment may also be a major driver limiting the success of the many clinical trials discussed above. The effect of pre-allogeneic hematopoietic stem cell transplantation conditioning intensity, which may predict the different likelihoods and impacts of residual host marrow cells and relevant pathobiologies described above, for *TP53*-mutated MDS/AML requires dedicated study. It is also unclear if the described mechanisms can at least partly be reproduced in neoplasms other than MDS/AML and similarly require further study.

Translational studies aimed at understanding the mechanisms of resistance and immune evasion by *TP53*-mutated leukemic cells are helping to guide future drug targets and trials. Amongst the most promising opportunities for effectively targeting the mutant *TP53* BMME are acting upon the p53 axis itself, the immunosuppressive or myeloproliferative cell-surface proteins, perturbed signaling pathways, and metabolic pathways upon which *TP53*-mutated MDS/AML pathobiology depends. The study of how these agents can best be harnessed to overcome the unique BMME in *TP53*-mutated disease is essential to drive better outcomes for patients.

## Figures and Tables

**Table 1:** Agents currently being evaluated in preclinical or clinical trial settings.

Therapy Type	Target	Drug	Phase	Trial Number	Result
p53 “targeted”	MDM2	Idasanutlin Alrizomadlin	Phase III Phase I/II	NCT02545283 NCT04358393	Terminated, futility Recruiting
	p53 “refolding”	Eprenetapopt APR-548	Phase III Phase I	NCT03745716 NCT04638309	Completed, no results Terminated, per sponsor
		Arsenic trioxide	Phase III	NCT03377725	Withdrawn
Immune checkpoint inhibitors	PD-L1	Pembrolizumab Nivolumab	Phase II Phase II Phase II/III	NCT04214249 NCT04284787 NCT03092674	Completed, no results Terminated, mortality Terminated, mortality
	PD-1	Durvalumab	Phase II	NCT02775903	Completed, no benefit
	CTLA-4	Ipilimumab	Phase II	NCT02530463	Completed
	TIM-3	Sabatolimab	Phase III	NCT04266301	Terminated, futility
	LAG-3	Retlatimab	Phase I/II	NCT04913922	Recruiting
	Clever-1	Bexmarilimab	Phase I/II	NCT05428969	Active, not recruiting
	LILRB4	IO-202 STAR-T	Phase I Phase I	NCT04372433 NCT05548088	Completed Completed
Other cell -surface protein targets	CD47	Magrolimab	Phase III Phase III Phase III	NCT04313881 NCT04778397 NCT05079230	Terminated, futility
	CD33	GO CD33-GSPT1 CAR T-cells CAR NK-cells	Phase III Preclinical Phase I/II Preclinical	NCT00927498 N/A NCT05984199 N/A	FDA approved N/A Terminated, funding N/A
	CD123 CD123-CD3 CD123-CD16	Talacotuzumab IMGN632 CAR T-cells CAR NK-cells Flotetuzumab Vibecotamab AFM28	Phase II/III Phase I/II Phase I Phase I Phase I/II Phase II Preclinical	NCT02472145 NCT03386513 NCT04230265 NCT02944162 NCT02152956 NCT05285813 N/A	Completed, no benefit Active, not recruiting Active, not recruiting Safety data only Completed, no benefit Completed N/A
Signal transduction pathways	Hedgehog	Glasdegib	Phase III	NCT03416179	Completed, no benefit
	Wnt	PRI-724	Preclinical	N/A	N/A
	NF-kB	Bortezomib	Phase III	NCT01371981	Completed, no benefit
	CCRL2	Anti-CCRL2-PBD	Preclinical	N/A	N/A
	HIF-1α CXCR4	Echinomycin Plerixafor	Preclinical Phase I	N/A NCT01352650	N/A Completed
Biologic pathways	HMG-CoA reductase	Pitavastatin HI-statins ^1^	Phase I Phase II	NCT04512105 NCT05483010	Safety data only Recruiting
	Glycolysis	N/A	Preclinical	N/A	N/A

^1^ HI-statins: atorvastatin and rosuvastatin.

## Data Availability

Data are contained within the article.

## Abbreviations

The following abbreviations are used in this manuscript:

BMME	Bone marrow microenvironment
AML	Acute myeloid leukemia
MDS	Myelodysplastic syndrome
CAR-T	Chimeric antigen receptor T-cell
BiTE	Bispecific T-cell engager
ADC	Antibody-drug conjugate
ICI	Immune checkpoint inhibitor
HPSC	Hematopoietic stem cell
MDSC	Myeloid-derived suppressor cell
